# Contribution of home garden vegetables on reducing stunting among 6‐ to 23‐month‐old children in South Tigray, northern Ethiopia

**DOI:** 10.1002/fsn3.3435

**Published:** 2023-05-22

**Authors:** Hailemariam Tekie Mahari, Zenebe Abraha Kahsay, Girmay Gebresamuel Abraha, Amanuel Zenebe Abraha, Carol J. Henry, Michael T. Nickerson, Afework Mulugeta Bezabih

**Affiliations:** ^1^ Department of Food Science and Post‐Harvest Technology Mekelle University Mekelle Ethiopia; ^2^ Department of Agricultural and Resources Economics Mekelle University Mekelle Ethiopia; ^3^ Department of Land Resource Management and Environmental Protection Mekelle University Mekelle Ethiopia; ^4^ Institute of Climate and Society Mekelle University Mekelle Ethiopia; ^5^ College of Pharmacy and Nutrition University of Saskatchewan Saskatoon Saskatchewan Canada; ^6^ Department of Food and Bioproduct Sciences University of Saskatchewan Saskatoon Saskatchewan Canada; ^7^ School of Public health, College of Health Sciences Mekelle University Mekelle Ethiopia

**Keywords:** children, difference‐in‐difference, non‐producer, stunting, vegetable producer

## Abstract

The study was conducted to analyze the contribution of home garden vegetables in reducing stunting among 6‐ to 23‐month‐old children from South Tigray, Northern Ethiopia. The quasi‐experimental study design was used. Multistage sampling technique was used to select the districts and study communities. A total of 94 purposively selected vegetable producer (intervention) households and 260 randomly selected non‐producer (control) households were included in the study (1:3 ratio). The recumbent length of children was measured using horizontal wooden board to the nearest 0.1 cm. The length‐for‐age *Z*‐scores were computed using WHO‐Anthro 2006 software. Propensity score‐matching and difference‐in‐difference (DID) estimates were used to analyze data using STATA software version 12. Prevalence of child stunting was 19.8 (12.7–29.4) and 21.1 (16.4–26.7)% (baseline) and 43.5 (33.5–54.1) and 46.5 (45.7–47.2)% (end line) among intervention and control groups, respectively. Child stunting was higher for boys and older children from both intervention and control households. DID estimation revealed that there was no significant difference in child stunting between intervention and control households (DID = 1.7, *p* = .604). However, there was an intervention effect of −0.5, 2.5, and 1.7% in the prevalence of child stunting among females, males, and both sexes, respectively. Vegetable production as an intervention strategy reduced the prevalence of stunting in children aged 6–23 months. However, vegetable production needs to be well integrated with other nutrition‐sensitive interventions to realize the objective of reducing child stunting.

## Graphical abstract

The study was conducted to analyze the contribution of home garden vegetables in reducing stunting in 6‐ to 23‐month‐old children from South Tigray, Northern Ethiopia, using a quasi‐experimental study. A total of 94 purposefully selected vegetable producer (intervention) households and 260 randomly selected non‐producer (control) households were included in the study (1:3 ratio). Although it was not significant, difference‐in‐difference estimation revealed that there was a positive intervention effect of home garden vegetables on child stunting (DID = 1.7, *p* = .604). Vegetable production as an intervention strategy reduced prevalence of stunting in children aged 6–23 months. However, vegetable production needs to be well integrated with other nutrition‐sensitive interventions to realize the objective of reducing child stunting.

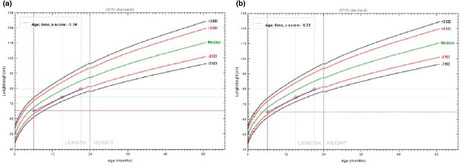

## 1 INTRODUCTION

Stunting (low height for age) is the result of long‐term chronic consumption of a low‐quality and less diverse diet in combination with morbidity, infectious diseases, and environmental problems (Haile et al., 2016; WHO, 2014). Childhood stunting is associated with adverse functional consequences including child mortality, poor cognition, low educational performance, low adult wages, poor socioeconomic development, and poor reproductive outcomes (Haile et al., 2016; UNICEF, 2013). Moreover, stunting reflects nutritional deficiencies during the most critical periods of growth and development in early life (Moges et al., 2015). In countries where food availability and access to nutrient‐rich food groups are severely restricted, the nutritional status of already vulnerable children is extremely concerning, with one in two children stunted (FSIN, 2020). In sub‐Saharan Africa (SSA), 34% of children aged less than 5 years are stunted, which is significantly higher than the global average of 21.3% (FAO, IFAD, UNICEF, WFP, WHO, 2017). About 49% of children aged under 5 years in Tigray are stunted, which is much higher than the average prevalence of child stunting (37%) in Ethiopia (CSA (Central Statistical Agency of Ethiopia), 2019).

Dietary intake is one of the risk factors for child stunting (WHO, 2004, 2011). Low fruit and vegetable consumption was listed as one of the top three diet‐related risks accounting for global deaths in 2000 (Lock et al., 2005). Studies conducted in Ethiopia (Moges et al., 2011) and India (Chauhan, 2015) revealed that dietary diversity is negatively associated with child stunting. Vegetables are essential components of a well‐balanced diet since they supply an abundant and inexpensive source of energy, body‐building nutrients, vitamins, minerals, dietary fiber, and phytochemicals (Dias, 2012; FAO, 2020; Hunde, 2017). Vegetables are also excellent resources for overcoming micronutrient deficiencies as well as providing smallholder farmers with much higher incomes and more jobs per hectare than staple crops (Davey et al., 2009).

Despite the nutritional and health benefits and the comparative advantage of a favorable climate in Ethiopia, particularly in the Tigray region, production and consumption of fruits and vegetables are very limited (Hunde, 2017; WHO, 2015). In Ethiopia, less than 20% of 6‐ to 23‐month‐old children received the minimum dietary diversity (FSIN, 2020). There are also inconsistencies in the effect of vegetable home gardening on the nutritional status of children due to differences in social, economic, and cultural issues. Thus, within the local context, identifying important gaps and generating knowledge on the effect of vegetable production on reducing stunting are imperative, which will help the design of better approaches to address context‐specific strategic interventions with home garden vegetables for improved nutritional status of children aged 6–23 months. The research was, therefore, conducted to determine the effect of home garden vegetables on reducing stunting in children aged 6–23 months.

## 2 METHODOLOGY

### 2.1 Study area

The study was conducted in Raya‐Azebo and Emba‐Alaje districts of the Southern zone of the Tigray region, Ethiopia. Five villages, namely Genete and Tsigea from Raya‐Azebo district and Ayba, Atsela, and Tek'a from Emba‐Alaje district, representing lowland and highland agroecological conditions, respectively, were selected and included in the study. The study areas were purposefully selected with the help of local experts based on better experiences in home garden vegetable production.

### 2.2 Study design

**FIGURE 1:**
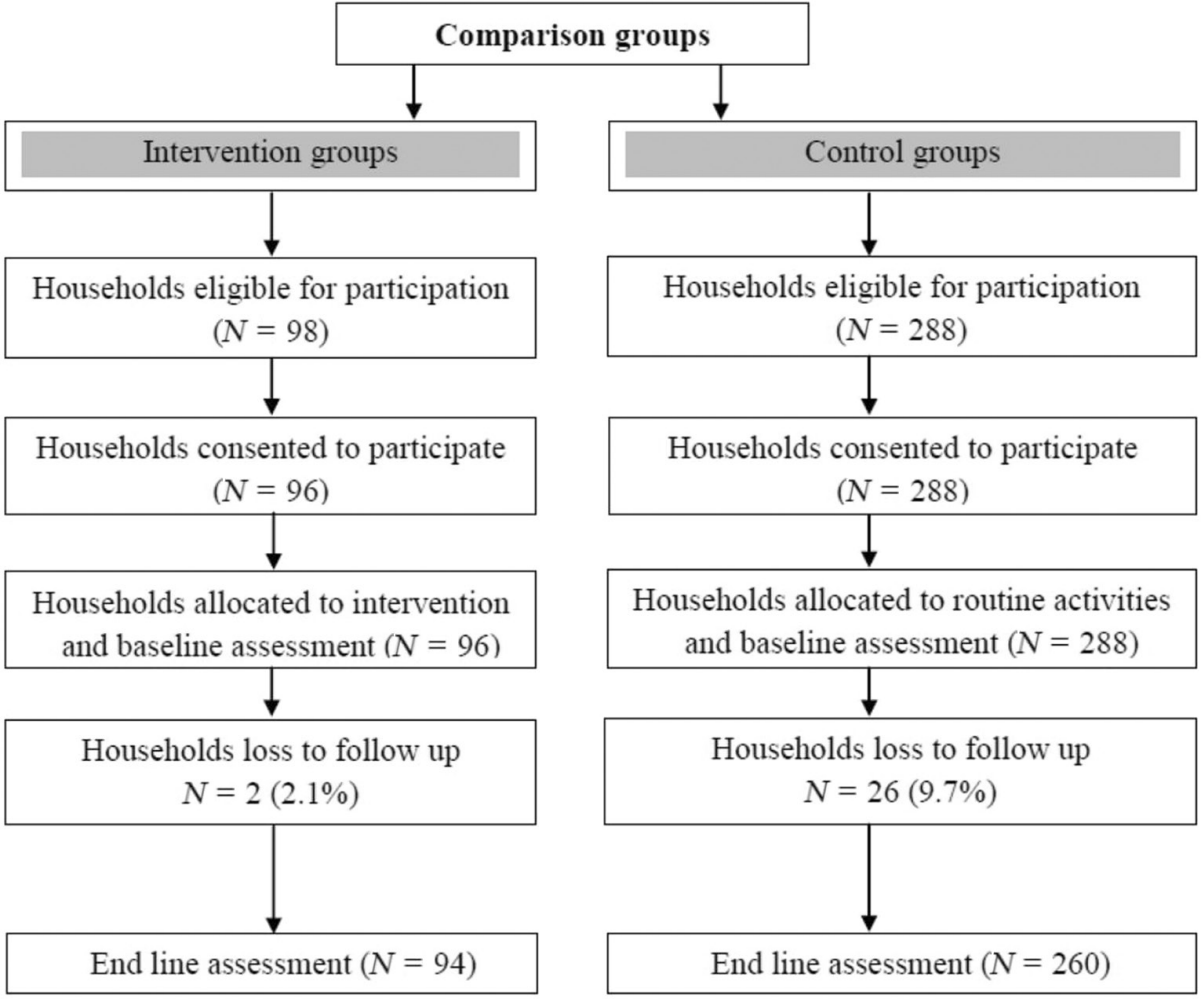
Schematic representation of the study design.

The study was conducted using a quasi‐experimental design to analyze the effect of home garden vegetable production in reducing child stunting. The schematic representation of the study design is shown in Figure 1.

### 2.3 Sample size and sampling technique

A total of 96 vegetable‐producer households and 288 non‐producer households were included in the study using the formula provided in the Food and Nutrition Technical Assistance III Sampling Guide**.**

$$n={\left[\left(\mathrm{Z\alpha}+\mathrm{Z\beta}\right)\;2^{*}\;\left\{\text{P1}\;\left(1-\text{P1}\right)+\text{P2}\;\left(1-\text{P2}\right)\right\}/\left(\text{P2}-P1\right)2\right]}^{*}\;D^{*}\;\mathrm{Nf}$$

*n* = required minimum sample size per survey round or comparison group; P1 = stunting rate at baseline, 39.3% = **0.39** (EDHS, 2016); P2 = the expected level of stunting at end line for the program area such that the quantity (P2 − P1) is the size of the magnitude of change it is desired to be able to detect, considering target reduction of 10 percentage points, 29% = **0.29**; *Zα* = the *Z*‐score corresponding to the degree of confidence with which it is desired to be able to conclude that an observed change of magnitude (P2 − P1) would not have occurred by chance (α—the level of statistical significance for one‐tailed test), 95% = **1.645**; *Zβ* = the *Z*‐score corresponding to the degree of confidence with which it is desired to be certain of detecting a change of magnitude (P2 − P1) if one actually occurred (β—statistical power), 80% = **0.840**; *D* = design effect for stunting = **1.0**; Nf = Non‐response factor (assuming a 5% non‐response rate) = **1.05**.

Based on the formula, the estimated target sample size (*n*) per comparison group is as follows:

$$n={\left[\left(1.645+0.84\right)2^{*}\;\left\{0.39\left(1-0.39\right)+0.29\left(1-0.29\right)\right\}/\left(0.29–0.39\right)2\right]}^{*}\;1.0^{*}\;1.05$$

*n* = 287.76 ≈ 288 per comparison group.

The intervention households were limited to 96 because it was not possible to get more than 96 households with access to home gardening and children 6 months of age. Hence, all households in the intervention group were included. Therefore, a total sample size of 384 (288 control and 96 intervention households) was used for the study.

Multistage sampling method was used to identify study districts and villages. Vegetable producer households were purposefully selected, while vegetable non‐producer households were randomly selected from the list of the total number of eligible households identified during the household listing exercises. Respondents were mothers or caretakers of the target households.

### 2.4 Description of the intervention

Households with a child 6 months of age and access to home gardening were included as intervention groups in the study. The selected households were supported by the development agents (DAs) and the researcher to grow a variety of vegetables. Participants in the intervention groups received training on vegetable production management. They were also provided training focusing on child nutrition using the nutrition education guidelines outlined by Hailu et al. (2016) and FAO (2009) to convey the messages on: child stunting, child and maternal nutrition problems, growing and consuming diverse and nutritious vegetables, infant and young child feeding practices, and maternal nutrition with the help of the Health Extension Workers (HEW). The training was provided twice in two production seasons to the intervention groups. Brochures were also provided to the training participants. Vegetable seeds of Swiss chard, carrots, lettuce, cabbage, beetroot, and tomato were distributed to the intervention groups as per their request and based on the agroecological condition of the study areas. The intervention considered both female‐ and male‐headed households. There was a close follow‐up by the local development agents (DAs) and HEWs and supervision by the principal investigator. The intervention was carried out for 15 months (December 2018–June 2020).

### 2.5 Data collection

Data on demographic and socioeconomic variables and anthropometrics were collected during the study period to evaluate the effect of home garden vegetables on child stunting. Except for the anthropometric measurements, all data were collected through face‐to‐face interviews using a structured questionnaire, which was administered in two phases: at the beginning and end of the intervention.

### 2.6 Anthropometric measurements

Recumbent length was collected three times during the study period (6‐, 15‐, and 21‐month‐old children). The length of a child was measured using a horizontal wooden length board in the recumbent position and read to the nearest 0.1 cm. The equipment needed to measure the length was placed on a flat surface, and the measurement was conducted by trained HEW as per the standard techniques of anthropometric measurements.

### 2.7 Data processing and analysis

Length for age of a child was expressed as a standard deviation unit from the median for the reference group and was compared to the 2006 WHO growth standards. Children who fall below −2 SD from the median of the standard population were regarded as moderately stunted, while those who fall below −3 SD from the median of the standard population were considered severely stunted. Children were, therefore, categorized by their length‐for‐age *Z*‐score (LAZ).

Descriptive statistics was used to determine the frequencies and percentages of the sociodemographic characteristics of the study participants. The length‐for‐age *Z*‐scores were computed using the WHO Anthro 2006 software. A propensity score matching (PSM) *t*‐test was conducted to check the significance level of the variables at baseline between the control and intervention groups. Difference‐in‐difference (DID) estimate was used to assess the effect of home garden vegetables on child stunting. An independent t‐test was performed using the gain scores of children. Analysis was done using tools in the STATA software version 12 at a statistical significance level of 5%.

## 3 RESULTS

### 3.1 Sociodemographic characteristics

The mean ages of the women respondents from vegetable producer and non‐producer households were 36.93 (95% CI: 35.71–38.15) and 35.35 (95% CI: 34.47–36.23) years, respectively. Most of the households producing vegetables were headed by men (97%). The mean family size of the producer households was 5.43 (95% CI: 5.16–5.71), while that of the non‐producer households was 4.92 (95% CI: 4.75–5.09; Table 1).

**TABLE 1:** Sociodemographic characteristics of the study participants.

Description	VP (*N* = 94)	VNP (*N* = 260)
	*n* (%)	*n* (%)
Villages		
Atsela	15 (16.0)	58 (22.3)
Tek'a	23 (24.5)	44 (16.9)
Ayba	22 (23.4)	46 (17.7)
Genete	16 (17.0)	52 (20.0)
Tsigea	18 (19.1)	60 (23.1)
Age of HH Head		
<25	5 (5.3)	23 (8.8)
25–35	44 (46.8)	146 (56.2)
36–55	43 (45.7)	82 (31.5)
>55	2 (2.1)	9 (3.5)
Sex of HH Head		
Male	91 (96.8)	231 (88.8)
Female	3 (3.2)	29 (11.2)
Family size		
<6	53 (56.4)	172 (66.2)
6 and above	41 (43.6)	88 (33.8)
Religion of HH Head		
Muslim	2 (2.1)	22 (8.5)
Orthodox Christian	92 (97.9)	238 (91.5)
Marital status		
Single	1 (1.1)	4 (1.5)
Married	92 (97.9)	236 (90.8)
Divorced	1 (1.1)	12 (4.6)
Widowed	0 (0.0)	8 (3.1)
Education of HH Head		
No education	37 (39.4)	103 (39.6)
Primary	55 (58.5)	154 (59.2)
Secondary	2 (2.1)	3 (1.2)
Education of spouse		
No education	45 (47.9)	137 (52.7)
Primary	46 (48.9)	122 (46.9)
Secondary	3 (3.2)	1 (0.4)
Vegetable consumption		
Yes	78 (83.0)	185 (71.2)
No	16 (17.0)	75 (28.8)
Source of livelihood		
Farming	86 (91.5)	182 (70.0)
Non‐farming	1 (1.1)	49 (18.8)
Both sources	7 (7.4)	29 (11.2)

Abbreviations: HH, household; VNP, vegetable non‐producers; VP, vegetable producers.

### 3.2 Prevalence of stunting among 6‐ to 23‐month‐old children

**FIGURE 2:**
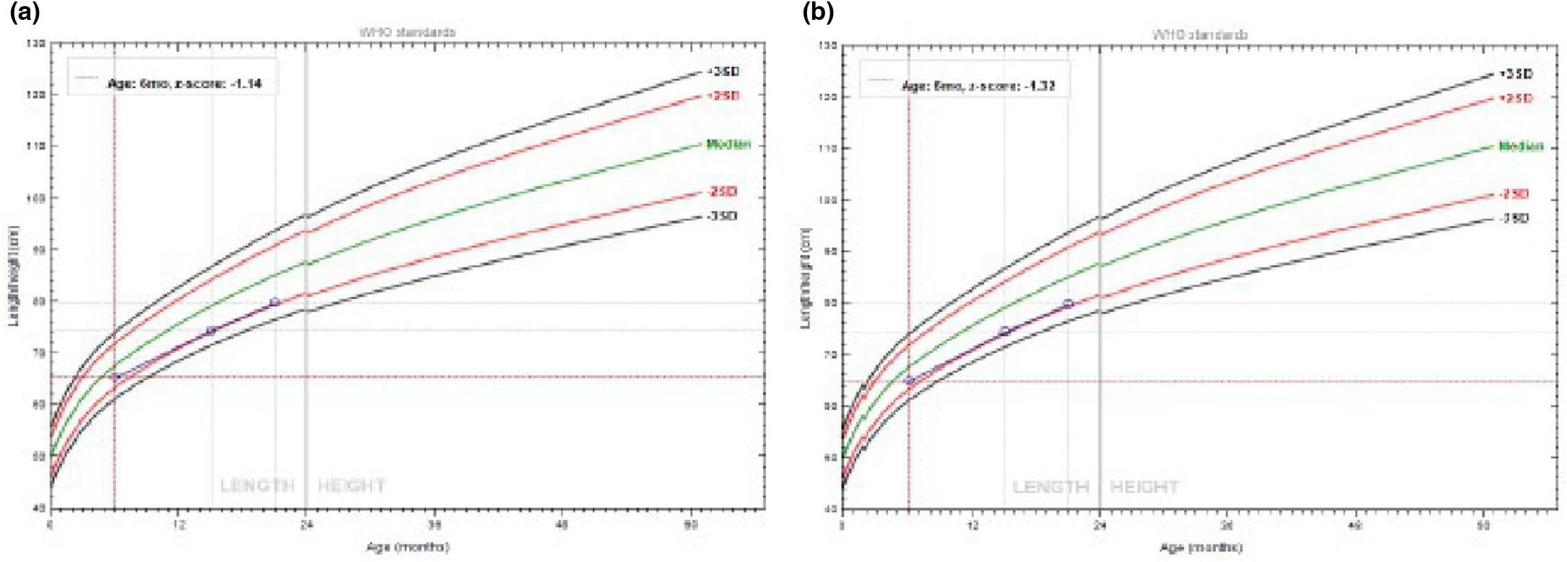
LAZ of children for vegetable producers (a) and non‐producers (b) over the study period (6, 15, and 21 months).

The prevalence of stunting was 19.8 (12.7–29.4) and 21.1 (16.4–26.7)% at baseline and 43.5 (33.5–54.1) and 46.5 (45.7–47.2)% at the end line among children from vegetable producer and non‐producer households, respectively. The prevalence of child stunting was higher in male compared to female children from both vegetable‐producer and non‐producer households (Table 2). Similarly, child stunting was found to be higher for older children in both vegetable‐producer and non‐producer households. The trend of child growth at the age of 6–23 months was, however, good in both groups in terms of their length‐for‐age *Z*‐score (Figure 2). The length‐for‐age *Z*‐score for vegetable producers at 6, 15, and 21 months was −0.73 ± 1.44, −1.58 ± 1.26, and −1.65 ± 1.76, respectively. Similarly, −0.83 ± 1.65, −1.47 ± 1.49, and −1.50 ± 1.63 were the *Z*‐scores at 6, 15, and 21 months for vegetable non‐producers, respectively.

**TABLE 2:** Prevalence of stunting among vegetable‐producing and non‐producing households.

Prevalence of stunting (%)	Vegetable producers						Vegetable non‐producers					
	Baseline			End line			Baseline			End line		
	Female	Male	Total	Female	Male	Total	Female	Male	Total	Female	Male	Total
Severe (<−3 *Z*‐scores)	0.0 (0.0–9.0)	14.9 (7.4–27.7)	8.1 (4.0–15.9)	15.8 (7.4–30.4)	29.8 (18.7–44.0)	23.5 (15.8–33.6)	4.9 (2.3–10.3)	20.0 (13.7–28.2)	12.2 (8.7–17.0)	12.1 (7.9–18.0)	27.4 (20.1–36.1)	19.5 (15.7–23.9)
Moderate (−3 to −2 *Z*‐scores)	12.8 (5.6–26.7)	10.6 (4.6–22.6)	11.6 (6.4–20.1)	26.3 (15.0–42.0)	14.9 (7.4–27.7)	20.0 (12.9–29.7)	6.6 (3.4–12.4)	11.3 (6.7–18.4)	8.9 (5.9–13.2)	28.2 (20.2–37.9)	25.6 (18.6–34.2)	27.0 (22.4–32.1)
Total (<−2 *Z*‐scores)	12.8 (5.6–26.7)	25.5 (15.3–39.5)	**19.8 (12.7–29.4)**	42.1 (27.9–57.8)	44.7 (31.4–58.8)	**43.5 (33.5–54.1)**	11.5 (7.0–18.3)	31.3 (23.5–40.3)	**21.1 (16.4–26.7)**	40.3 (36.4–44.3)	53.0 (44.0–61.8)	**46.5 (45.7–47.2)**

Bold values indicate the magnitude of child stunting prevalence.

### 3.3 Difference‐in‐differences estimation results

Kernel PSM *t*‐test of the control and intervention groups at the baseline showed that there was no significant difference between the groups for the outcome variable with covariates (Table 3). As the PSM *t*‐test of the variables was insignificant at the baseline, the effect of the intervention was determined using DIDs estimate.

**TABLE 3:** Kernel propensity score matching (PSM) *t*‐test at baseline.

Weighted variable(s)	Control	Treated	Diff.^ns^
Prevalence of stunting	1.189	1.186	−0.003
Sex of HHH	0.972	0.965	−0.007
Age of HHH	36.174	35.953	−0.221
Marital status of HHH	0.974	0.977	0.003
Education status of HHH	1.620	1.628	0.008
Education status of spouse	1.527	1.547	0.020
Family size	5.515	5.453	−0.061
Land size	0.770	0.873	0.103
Livestock production	0.852	0.847	−0.005
Logincome	4.145	4.160	0.015
Child vitamin A supplements	0.680	0.709	0.030
Child food supplements	0.299	0.291	−0.008
Nutrition training (last year)	0.355	0.349	−0.006
Bottle feeding	0.085	0.105	0.020
Complementary feeding	0.321	0.357	0.036
HDDS	6.841	6.860	0.019
HFIAS	1.807	1.753	−0.054

ns ‐ non‐significant.

Table 4 shows that there was a difference of 1.3 and 3.0% in the prevalence of child stunting between the control and intervention groups at the baseline and endline surveys, respectively, with a higher prevalence of child stunting for the control groups (vegetable non‐producer households). DIDs estimation results revealed that there was no significant difference between female (*p* = .579) and male children (*p* = .775) in control and intervention groups based on the prevalence of stunting (Table 4). The difference observed in the prevalence of child stunting due to the intervention effect was only −0.5% and 2.5% for female and male children, respectively (Table 4). Similarly, there was no significant difference for all children (both sexes; *p* = .604) by which a difference of 1.7% in the prevalence of child stunting was observed between the intervention and control groups over the study period.

**TABLE 4:** Difference‐in‐difference estimation results based on prevalence of child stunting (%).

Prevalence of stunting (%) (95% CI)	Vegetable producer (*n* = 94)			Vegetable non‐producer (*n* = 260)			Intervention effect (DID estimates)	*p*‐value
	Baseline	End line	Diff (%)	Baseline	End line	Diff (%)		
Female	12.8 (5.6–26.7)	42.1 (27.9–57.8)	29.3	11.5 (7.0–18.3)	40.3 (36.4–44.3)	28.8	−0.5	.579
Male	25.5 (15.3–39.5)	44.7 (31.4–58.8)	19.2	31.3 (23.5–40.3)	53.0 (44.0–61.8)	21.7	2.5	.775
Total	**19.8 (12.7–29.4)**	**43.5 (33.5–54.1)**	23.7	**21.1 (16.4–26.7)**	**46.5 (45.7–47.2)**	25.4	1.7	.604

Bold values indicate p‐values are less than 0.05.

## 4 DISCUSSION

In this study, the sex and age of the children were identified as determinant factors of the length for age of children in the age range 6–23 months. Similar findings were reported where being a male child (Berhanu et al., 2018; Ntenda & Chuang, 2018; Yaya et al., 2020) and children in higher age groups (Alemayehu et al., 2020; Geberselassie et al., 2018; Getaneh et al., 2019) increased the odds of child stunting. According to Thurstans et al. (2021) and Bork and Diallo (2017), the higher prevalence of stunting in male children is due to the lower immune response and capacity to produce antibodies in boys to cause higher vulnerability to infectious and non‐communicable diseases. *The difference in the* hormonal systems between female and male children also causes boys to require relatively more calorie‐dense foods for proper growth (Merkiel‐Pawłowska & Chalcarz, 2017; Saville et al., 2021). Moreover, the prevalence of stunting in male children is higher in cultures, particularly in SSA, in which girls enjoy a nutritional premium relative to boys (Mussa, 2015; Mwale et al., 2020). As a culture, female children have more contact times with their mothers so they are more protected than male children in the study areas. Hence, this might have contributed to the higher prevalence of stunting in male children.

Furthermore, children might be exposed to health problems due to poor caring practices when they grow up. This was supported by Batiro et al. (2017) and Teshome et al. (2009) who observed a gap in nutritional requirements with increasing age of a child, which in turn causes limited growth of children. Besides, Gelu et al. (2018) indicated that when a child is put on a family diet he suffers from a lack of adequate dietary diversity and meal frequency which is also commonly observed in Ethiopia. On the other hand, Teferi et al. (2016), in a study from Shey Bench district, in Southwest Ethiopia, reported a contrasting result where the highest risk of stunting occurred in the youngest age group. Gebre et al. (2019) found the highest prevalence of stunting among children aged 12–23 months and the lowest prevalence in children aged 48–59 months. This indicates that stunting may occur inconsistently regardless of age group which confirms the need for context‐specific studies.

The trend of child growth based on the length‐for‐age *Z*‐score was similar in both the control and intervention groups during the study. Although significant difference was not observed, DID estimation results revealed that the prevalence of child stunting was reduced by 1.7% due to vegetable production compared to vegetable non‐producers. This indicates that vegetable production could improve the chance of reducing child stunting and, thus, more effort is needed to significantly and positively affect the length‐for‐age *Z*‐score of children. According to Baliki et al. (2019), the effect of vegetable consumption at the beginning was not statistically different from consumption 1 year later, which demonstrates that impact was observed in the long term. The most important determinants of children's vegetable intake are age of the child, social and cultural norms, economic status, preferences, parental intake, and home availability/accessibility (Jeong & Lee, 2021; Kehoe et al., 2019; Raggio & Gámbaro, 2018). Parents, especially mothers, influence children's vegetable preferences and the majority of them mentioned the sensory characteristics of vegetables as the main determinant of children's vegetable consumption (Kral & Faith, 2009; Laureati, 2022; Mahmood et al., 2021). Sensory attributes such as bitter taste, green color, texture, and appearance were reported as drivers for the rejection of vegetable consumption in children (Estay et al., 2019; Mustonen et al., 2012). Hence, the lower impact of vegetables on the prevalence of child stunting in the study areas could be due to the dislike of vegetables by children or the limited previous experience of vegetable consumption at the household level and other economic and social factors. As children develop preferences over time and learn via experiences with food and eating (Hetherington et al., 2011; Schwartz et al., 2011), different researchers (Anzman‐Frasca et al., 2012; Caton et al., 2013; Estay et al., 2021) suggested a repeated exposure and culinary strategies to modify some of these sensory characteristics as an effective method of promoting vegetable intake in young children.

It is believed that home gardening can directly enhance household food security and then nutritional status by providing access to a diversity of nutritionally‐rich foods, increased purchasing power from savings on food bills and income from sales of garden products, and fallback food provision during seasonal lean periods (FAO and FHI 360, 2016). Despite these facts, there are reports of increased child stunting in households having access to home gardening which could be due to limited knowledge about which foods should be consumed to address macro‐ and micronutrient deficiencies for the life span, and hence underestimate what is needed for children (Beyene et al., 2019). However, in this study, it is only due to the vegetable intervention that the prevalence of child stunting was reduced by 1.7% depicting that more reduction in the prevalence of child stunting could be achieved if vegetable intervention is integrated with other nutrition‐sensitive interventions (e.g., animal source foods).

## 5 CONCLUSION

There is a high prevalence of child stunting in the study areas but the status of child stunting is different for different sex and age groups of children, where it was highest for boys and older children. Vegetable production as an intervention strategy reduced the prevalence of stunting in children aged 6–23 months. The availability of vegetable production is not enough for improving nutritional status of children. Hence, home garden vegetable production needs to be well integrated with the existing demographic and socioeconomic factors such as feeding practices to improve consumption of diverse and nutritious foods by the households, particularly children so as to realize the objective of reducing child stunting. Further study is also needed to investigate better design of strategic interventions of vegetables to address the problem of child stunting.

## AUTHOR CONTRIBUTIONS

**Hailemariam Tekie Mahari:** Conceptualization (equal); data curation (equal); formal analysis (equal); funding acquisition (equal); investigation (equal); methodology (equal); resources (equal); supervision (equal); visualization (equal); writing – original draft (equal); writing – review and editing (equal). **Zenebe Abraha Kahsay:** Conceptualization (equal); data curation (equal); formal analysis (equal); investigation (equal); methodology (equal); resources (equal); supervision (equal); visualization (equal); writing – original draft (equal); writing – review and editing (equal). **Girmay Gebresamuel Abraha:** Conceptualization (equal); investigation (equal); methodology (equal); resources (equal); supervision (equal); visualization (equal); writing – original draft (equal); writing – review and editing (equal). **Amanuel Zenebe Abraha:** Conceptualization (equal); data curation (equal); formal analysis (equal); funding acquisition (equal); investigation (equal); methodology (equal); project administration (equal); resources (equal); supervision (equal); visualization (equal); writing – original draft (equal); writing – review and editing (equal). **Carol J. Henry:** Funding acquisition (equal); resources (equal); writing – original draft (equal); writing – review and editing (equal). **Michael T. Nickerson:** Funding acquisition (equal); resources (equal); writing – original draft (equal); writing – review and editing (equal). **Afework Mulugeta Bezabih:** Conceptualization (equal); data curation (equal); formal analysis (equal); funding acquisition (equal); investigation (equal); methodology (equal); resources (equal); supervision (equal); visualization (equal); writing – original draft (equal); writing – review and editing (equal).

## FUNDING INFORMATION

This work was supported by the African Climate Change Adaptation Initiative project (ACCAI) of the Open Society Institute, Mekelle University, with grant No. OR2014‐18350 to conduct the research. The funder had no role in study design, data collection and analysis, decision to publish, or preparation of the manuscript.

## CONFLICT OF INTEREST STATEMENT

The authors declare that they have no conflict of interest.

## ETHICAL APPROVAL

Ethical clearance was obtained from the Research Ethics Review Committee (RERC) of the Institute of Climate and Society, Mekelle University. Moreover, the study was approved by the local offices at the district and sub‐district levels. After the approval from local officials, each participant consented orally to participate in the study and was informed of the objectives of the study, and their rights to refuse participation, stop participating at any time during the interview, and skip specific questions or topics they do not want to answer.

## Data Availability

The data that support the findings of this study are openly available in figshare at http://doi.org/10.6084/m9.figshare.22730066.

## References

[fsn33435-bib-0001] Alemayehu, F. R. , Gebre‐Mariam, R. , Loha, E. , Anato, A. , & Desta, D. T. (2020). Maternal socio demographic characteristics are associated with child stunting in Alamura subcity of Hawassa, Ethiopia. Public Health Research, 10(1), 12–20.

[fsn33435-bib-0002] Anzman‐Frasca, S. , Savage, J. S. , Marini, M. E. , Fisher, J. O. , & Birch, L. L. (2012). Repeated exposure and associative conditioning promote preschool children's liking of vegetables. Appetite, 58(2), 543–553. 22120062 10.1016/j.appet.2011.11.012

[fsn33435-bib-0003] Baliki, G. , Brück, T. , Schreinemachers, P. , & Uddin, M. N. (2019). Long‐term behavioural impact of an integrated home garden intervention: Evidence from Bangladesh. Food Sec., 11, 1217–1230.

[fsn33435-bib-0004] Batiro, B. , Demissie, T. , Halala, Y. , & Anjulo, A. A. (2017). Determinants of stunting among children aged 6–59 months at Kindo Didaye woreda, Wolaita zone, Southern Ethiopia: Unmatched case control study. PLoS One; 12 (12): e0189106. 29261680 10.1371/journal.pone.0189106 PMC5737969

[fsn33435-bib-0005] Berhanu, G. , Mekonnen, S. , & Sisay, M. (2018). Prevalence of stunting and associated factors among preschool children: A community based comparative cross sectional study in Ethiopia. BMC Nutrition, 4, 28. 32153889 10.1186/s40795-018-0236-9 PMC7050938

[fsn33435-bib-0006] Beyene, S. , Willis, M. S. , Mamo, M. , Legesse, B. , Regassa, T. , Tadesse, T. , Wolde‐Hawariat, Y. , & Roslan, N. F. (2019). Nutritional status of children aged 0–60 months in two drought‐prone areas of Ethiopia. South African Journal of Clinical Nutrition, 33(4), 152–157.

[fsn33435-bib-0007] Bork, K. , & Diallo, A. (2017). Boys are more stunted than girls from early infancy to 3 years of age in rural Senegal. The Journal of Nutrition, 147(5), 940–947. 28298540 10.3945/jn.116.243246

[fsn33435-bib-0008] Caton, S. J. , Ahern, S. M. , Remy, E. , Nicklaus, S. , Blundell, P. , & Hetherington, M. M. (2013). Repetition counts: Repeated exposure increases intake of a novel vegetable in UK pre‐school children compared to flavour–flavour and flavour–nutrient learning. British Journal of Nutrition, 109(11), 2089–2097. 23110783 10.1017/S0007114512004126

[fsn33435-bib-0009] Chauhan, R. (2015). An Investigation of the Relationships Among Homegardens, Dietary Diversity, and the Nutritional Status of Children Aged 0 to 5 in Indian Households (Doctoral dissertation, University of Illinois at Urbana‐Champaign).

[fsn33435-bib-0010] CSA (Central Statistical Agency of Ethiopia) . (2019). Ethiopia mini demographic and health survey 2019. Federal Democratic Republic of Ethiopia, Central Statistical Agency (CSA).

[fsn33435-bib-0011] Davey, M. W. , Van den Bergh, I. , Markham, R. , Swennen, R. , & Keulemans, J. (2009). Genetic variability in Musa fruit provitamin A carotenoids, lutein and mineral micronutrient contents. Food Chemistry, 115(3), 806–813.

[fsn33435-bib-0012] Dias, J. S. (2012). Nutritional quality and health benefits of vegetables: A review. Food and Nutrition Sciences, 3(10), 1354–1374.

[fsn33435-bib-0013] EDHS . (2016). Ethiopia Demographic and Health Survey 2016: key indicators report.

[fsn33435-bib-0014] Estay, K. , Pan, S. , Zhong, F. , Capitaine, C. , & Guinard, J. X. (2019). A cross‐cultural analysis of children's vegetable preferences. Appetite, 142, 104346. 31278955 10.1016/j.appet.2019.104346

[fsn33435-bib-0015] Estay, K. , Kurzer, A. , & Guinard, J. X. (2021). Mothers’ Perceptions and Attitudes towards Children’s Vegetable Consumption—A Qualitative, Cross‐cultural Study of Chilean, Chinese and American Mothers Living in Northern California. Foods, 10(3), 519. 33801450 10.3390/foods10030519 PMC8000429

[fsn33435-bib-0016] FAO . (2009). Manual: Growing vegetables for home and market. Diversification Booklet number 11.

[fsn33435-bib-0017] FAO . (2020). Fruit and vegetables – your dietary essentials. The International Year of Fruits and Vegetables, 2021, background paper. Rome. Retrieved from 10.4060/cb2395en

[fsn33435-bib-0018] FAO and FHI 360 . (2016). Minimum dietary diversity for women: A guide for measurement. Rome, Italy.

[fsn33435-bib-0019] FAO, IFAD, UNICEF, WFP, WHO . (2017). The state of food security and nutrition in the world 2017. In Building resilience for peace and food security. FAO.

[fsn33435-bib-0020] FSIN (Food Security Information Network) . (2020). *Global Report on Food Crises* (GRFC).

[fsn33435-bib-0021] Geberselassie, S. B. , Abebe, S. M. , Melsew, Y. A. , Mutuku, S. M. , & Wassie, M. M. (2018). Prevalence of stunting and its associated factors among children 6–59 months of age in Libo‐Kemekem district, Northwest Ethiopia; a community based cross sectional study. PLoS One, 13(5), e0195361. 29723280 10.1371/journal.pone.0195361 PMC5933689

[fsn33435-bib-0022] Gebre, A. P. , Reddy, S. , Mulugeta, A. , Sedik, Y. , & Kahssay, M. (2019). Prevalence of malnutrition and associated factors among under‐five children in pastoral communities of Afar regional state, Northeast Ethiopia: A community‐based cross‐sectional study. Journal of Nutrition and Metabolism, 2019, 1–13. 10.1155/2019/9187609 PMC6589243 31275645

[fsn33435-bib-0023] Gelu, A. , Edris, M. , Derso, T. , & Abebe, Z. (2018). Undernutrition and associated factors among children aged 6–59 months living in slum areas of Gondar city, Northwest Ethiopia: A cross‐sectional study. Journals Pediatric Health, Medicine and Therapeutics, 9, 81–88. 10.2147/PHMT.S172317 PMC6118270 30215624

[fsn33435-bib-0024] Getaneh, Z. , Melku, M. , Geta, M. , Melak, T. , & Hunegnaw, M. T. (2019). Prevalence and determinants of stunting and wasting among public primary school children in Gondar town, northwest, Ethiopia. BMC Pediatrics, 19, 207. 31238889 10.1186/s12887-019-1572-x PMC6591879

[fsn33435-bib-0025] Haile, D. , Azage, M. , & Mola, T. (2016). Exploring spatial variations and factors associated with childhood stunting in Ethiopia: Spatial and multilevel analysis. BMC Pediatrics, 16, 49. 27084512 10.1186/s12887-016-0587-9 PMC4833938

[fsn33435-bib-0026] Hailu, T. , Tessema, M. , Fofanah, M. , & Lema, Z. (2016). Nutrition training manual for health and agriculture workers at community level in Ethiopia: Integrating nutrition into crop and livestock farming systems of the Ethiopian highlands for improved nutrition outcomes. ILRI.

[fsn33435-bib-0027] Hetherington, M. M. , Cecil, J. E. , Jackson, D. M. , & Schwartz, C. (2011). Feeding infants and young children. From guidelines to practice. Appetite, 57(3), 791–795. 21784114 10.1016/j.appet.2011.07.005

[fsn33435-bib-0028] Hunde, N. F. (2017). Opportunity, problems and production status of vegetables in Ethiopia: A review research article. Plant Science, 4(2), 172.

[fsn33435-bib-0029] Jeong, S. , & Lee, J. (2021). Effects of cultural background on consumer perception and acceptability of foods and drinks: A review of latest cross‐cultural studies. Current Opinion in Food Science, 42, 248–256.

[fsn33435-bib-0030] Kehoe, S. H. , Dhurde, V. , Bhaise, S. , Kale, R. , Kumaran, K. , Gelli, A. , & Fall, C. H. (2019). Barriers and facilitators to fruit and vegetable consumption among rural Indian women of reproductive age. Food and Nutrition Bulletin, 40(1), 87–98. 30974984 10.1177/0379572118816459 PMC6660308

[fsn33435-bib-0031] Kral, T. V. , & Faith, M. S. (2009). Influences on child eating and weight development from a behavioral genetics perspective. Journal of Pediatric Psychology, 34(6), 596–605. 18407923 10.1093/jpepsy/jsn037 PMC13411772

[fsn33435-bib-0032] Laureati, M. (2022). Determinants of preference and consumption of healthy food in children. Food, 11(2), 203. 10.3390/foods11020203 PMC8775126 35053934

[fsn33435-bib-0033] Lock, K. , Pomerleau, J. , Cause, L. , Altmann, D. R. , & McKee, M. (2005). The global burden of disease attributable to low consumption of fruit and vegetables: Implications for the global strategy on diet. Bulletin of the World Health Organization, 83, 100–108. 15744402 PMC2623811

[fsn33435-bib-0034] Mahmood, L. , Flores‐Barrantes, P. , Moreno, L. A. , Manios, Y. , & Gonzalez‐Gil, E. M. (2021). The influence of parental dietary behaviors and practices on children's eating habits. Nutrients, 13(4), 1138. 33808337 10.3390/nu13041138 PMC8067332

[fsn33435-bib-0035] Merkiel‐Pawłowska, S. , & Chalcarz, W. (2017). Gender differences and typical nutrition concerns of the diets of preschool children—The results of the first stage of an intervention study. BMC Pediatrics, 17(1), 207. 29258537 10.1186/s12887-017-0962-1 PMC5735756

[fsn33435-bib-0036] Moges, T. , Birks, K. A. , Samuel, A. , Kebede, A. , Kebede, A. , Wuehler, S. , Zerfu, D. , Abera, A. , Mengistu, G. , Tesfaye, B. , & Addis, G. (2011). Diet Diversity is Negatively Associated with Stunting among Ethiopian Children 6–35 Months of Age.

[fsn33435-bib-0037] Moges, B. , Feleke, A. , Meseret, S. , & Doyore, F. (2015). Magnitude of stunting and associated factors among 6‐59 months old children in Hossana town, Southern Ethiopia. Journal of Clinical Research & Bioethics, 6, 207.

[fsn33435-bib-0038] Mussa, R. (2015). Intra‐household and inter‐household child nutrition inequality in Malawi. South African Journal of Economics, 83(1), 140–153.

[fsn33435-bib-0039] Mustonen, S. , Oerlemans, P. , & Tuorila, H. (2012). Familiarity with and affective responses to foods in 8–11‐year‐old children: The role of food neophobia and parental education. Appetite, 58, 777–780. 22326884 10.1016/j.appet.2012.01.027

[fsn33435-bib-0040] Mwale, M. L. , Kamninga, T. M. , & Cassim, L. (2020). Gender gaps in child nutrition in Malawi: Does cultural lineage matter? Emerald Open Research, 2, 9.

[fsn33435-bib-0041] Ntenda, P. A. M. , & Chuang, Y.‐C. (2018). Analysis of individual‐level and community‐level effects on childhood undernutrition in Malawi. Pediatrics and Neonatology, 59(4), 380–389. 29295806 10.1016/j.pedneo.2017.11.019

[fsn33435-bib-0042] Raggio, L. , & Gámbaro, A. (2018). Study of the reasons for the consumption of each type of vegetable within a population of school‐aged children. BMC Public Health, 18(1), 1–11. 10.1186/s12889-018-6067-4 PMC6173934 30290788

[fsn33435-bib-0043] Saville, N. M. , Harris‐Fry, H. , Marphatia, A. , Reid, A. , Cortina‐Borja, M. , Manandhar, D. S. , & Wells, J. C. (2021). Differences in maternal and early child nutritional status by offspring sex in lowland Nepal. American Journal of Human Biology, 34(3), e23637. 34228379 10.1002/ajhb.23637 PMC12086752

[fsn33435-bib-0044] Schwartz, C. , Chabanet, C. , Lange, C. , Issanchou, S. , & Nicklaus, S. (2011). The role of taste in food acceptance at the beginning of complementary feeding. Physiology & Behavior, 104(4), 646–652. 21554893 10.1016/j.physbeh.2011.04.061

[fsn33435-bib-0046] Teferi, M. B. , Hassen, H. Y. , Kebede, A. , Adugnaw, E. , Gebrekrstos, G. , & Guesh, M. (2016). Prevalence of stunting and associated factors among children aged 06–59 months in Southwest Ethiopia: A cross‐sectional study. Journal of Nutritional Health & Food Science, 4(6), 1–6.28944285

[fsn33435-bib-0047] Teshome, B. , Kogi‐Makau, W. , Getahun, Z. , & Taye, G. (2009). Magnitude and determinants of stunting in children under‐five years of age in food surplus region of Ethiopia. The case of west Gojam zone. Ethiopian Journal of Health Development, 23(2), 98–106.

[fsn33435-bib-0048] Thurstans, S. , Opondo, C. , Seal, A. , Wells, J. , Khara, T. , Dolan, C. , et al. (2021). Understanding sex differences in childhood undernutrition: A narrative review. Nutrients, 14(5), 948. 10.3390/nu14050948 PMC8912557 35267923

[fsn33435-bib-0049] UNICEF . (2013). Improving child nutrition: the achievable imperative for global progress (Vol. 1–16, pp. 56–67). United Nations Children’s Fund.

[fsn33435-bib-0050] WHO . (2004). Vitamin and mineral requirements in human nutrition. World Health Organization.

[fsn33435-bib-0051] WHO . (2011). Prevention of iron deficiency anaemia in adolescents: role of weekly iron and folic acid supplementation SEA‐CAH‐02. World Health Organization.

[fsn33435-bib-0052] WHO . (2014). Childhood stunting: Challenges and opportunities. Report of a promoting healthy growth and preventing childhood stunting colloquium. World Health Organization.

[fsn33435-bib-0053] WHO . (2015). Micronutrient deficiencies. Retrieved from http://www.who.int/nutrition/topics/ida/en/

[fsn33435-bib-0054] Yaya, S. , Oladimeji, O. , Odusina, E. K. , & Bishwajit, G. (2020). Household structure, maternal characteristics and children's stunting in sub‐Saharan Africa: Evidence from 35 countries. International Health, ihz105, 381–389. 10.1093/inthealth/ihz105 PMC9248065 31927593

